# Community-Wide Active Case Finding for Tuberculosis: Time to Use the Evidence We Have

**DOI:** 10.3390/tropicalmed9090214

**Published:** 2024-09-14

**Authors:** Mikaela Coleman, Chris Lowbridge, Philipp du Cros, Ben J. Marais

**Affiliations:** 1 Sydney Infectious Diseases Institute, University of Sydney, Sydney, NSW 2050, Australia; ben.marais@sydney.edu.au; 2 Bordeaux Population Health, University of Bordeaux, 33076 Bordeaux, France; 3 Division of Global & Tropical Health, Menzies School of Health Research, Charles Darwin University, Casuarina, NT 0810, Australia; christopher.lowbridge@menzies.edu.au; 4 International Health, Burnet Institute, Melbourne, VIC 3004, Australia; philipp.ducros@burnet.edu.au; 5 Department of Infectious Diseases, Monash Medical Centre, Clayton, VIC 3168, Australia; 6 WHO Collaborating Centre for Tuberculosis, Sydney, NSW 2145, Australia

**Keywords:** tuberculosis, active case finding, systematic screening, elimination, population-wide, community-wide, TB

## Abstract

Tuberculosis, caused by the *Mycobacterium tuberculosis* (*Mtb*) bacteria, is one of the world’s deadliest infectious diseases. Despite being the world’s oldest pandemic, tuberculosis is very much a challenge of the modern era. In high-incidence settings, all people are at risk, irrespective of whether they have common vulnerabilities to the disease warranting the current WHO recommendations for community-wide tuberculosis active case finding in these settings. Despite good evidence of effectiveness in reducing tuberculosis transmission, uptake of this strategy has been lacking in the communities that would derive greatest benefit. We consider the various complexities in eliminating tuberculosis from the first principles of the disease, including diagnostic and other challenges that must be navigated under an elimination agenda. We make the case that community-wide tuberculosis active case finding is the best strategy currently available to drive elimination forward in high-incidence settings and that no time should be lost in its implementation. Recognizing that high-incidence communities vary in their epidemiology and spatiosocial characteristics, tuberculosis research and funding must now shift towards radically supporting local implementation and operational research in communities. This “preparing of the ground” for scaling up to community-wide intervention centers the local knowledge and local experience of community epidemiology to optimize implementation practices and accelerate reductions in community-level tuberculosis transmission.

## 1. Introduction

*“A goal without a plan is just a wish”—Antoine de Saint-Exupéry*

*Mycobacterium tuberculosis* (*Mtb*), the causative agent of tuberculosis, is an ancient human pathogen [1] that may be linked to our unique ability to control fire [2]. Despite major advances in medicine, tuberculosis remains the world’s leading infectious disease killer, claiming an estimated 1.3 million lives and causing 10.6 million new cases in 2022 [3]. The complexity of the disease, its numerous manifestations, and the interplay of various risk factors are just some of the challenges that make tuberculosis a remarkably persistent human predator. If meaningful progress is to be made towards the 90% reduction in global tuberculosis incidence envisaged by the 2030 Sustainable Development Goal (SDG) targets and the World Health Organization’s (WHO) End TB strategy 2035 [4,5], then anti-tuberculosis efforts need to become radically more aggressive. In this review, we consider just a few of the many host and pathogen factors that contribute to the persistence of tuberculosis and make the case that population-based active tuberculosis case finding is a strategy that we already have to neutralize these challenges in high-incidence settings.

### 1.1. Evolving Tuberculosis Diagnostics and Treatments

As an airborne infection, *Mtb* is transmitted when small bacilli-containing aerosols are produced by an infectious person through coughing, breathing, laughing, talking, and singing and deposited in the distal airways through inhalation [6,7,8]. Host and pathogen factors determine whether an exposed person progresses to disease, establishes latent infection, or self-resolves [8]. In the absence of a non-human reservoir, it is human-to-human transmission that sustains the global epidemic. Human-targeted interventions seek to interrupt this transmission, primarily through early diagnosis and prevention, yet high disease incidence rates continue to prevail in most countries worldwide (92 countries have incidence > 50 cases per 100,000 people) [9]. Tools with which to detect and effectively treat tuberculosis have been available since the mid-20th century. The *Bacillus Calmette-Guérin* (BCG) vaccine, with a 12–74% age-dependent protective effect against tuberculosis [10,11], was first delivered in 1921 [12]. The quest for a more protective vaccine is ongoing, but the process is challenging given the delicate immune balance that has evolved over thousands of years [13]. Diagnostics such as chest X-ray and/or sputum smear microscopy together with effective antibiotic drug regimens to treat active disease and latent *Mtb* infection were used throughout the 1950s and 1960s to help eliminate tuberculosis in high-income countries like the United Kingdom, Australia, and the United States [14,15,16,17,18,19]. Improved living conditions and management of risk factors in these countries also contributed to the decline, suggesting that active tuberculosis interventions combined with reductions in risk-factors can have a population-level epidemiological impact that drives disease rates towards elimination [20,21].

Despite these mid-century successes, these diagnostic tools were often not fit for purpose in under-resourced and remote settings. Similarly, treatment regimens for both disease and latent infection were oppressively long (1–3-years and 6-months, respectively) [22]. In consequence, the value of these tools for large-scale active case finding (ACF) in high-incidence settings was not seriously evaluated until the 21st century brought diagnostic advances [23,24]. Today, the ultra-portable digital X-ray with computer aided detection (CAD) software minimizes the challenge of radiographic diagnosis in hard-to-reach communities. Cartridge-based PCR systems using sputum [25,26], stool [27], nasopharyngeal aspirate [28], and blood [29] samples—most notably the GeneXpert^®^ system—allow for microbiological confirmation of smear-positive and -negative diseases [30], along with resistance testing at room temperature, with minimal reagent and electricity needs and a rapid result turn-around. Emergent biomarker [31], urine LAM [32], and other point-of-care technologies to aid diagnosis amongst children and those unable to produce sputum are also increasingly feasible [33]. The development of tests with high diagnostic yield increases the population coverage of testing, and is an equally important consideration as accuracy and specificity when choosing tests for use in population-based screening (considered more comprehensively by others) [34]. Finally, the development of shorter, better-tolerated treatment regimens promote treatment completion [35,36]. Tolerability of treatment is particularly valuable for latent *Mtb* infection and subclinical tuberculosis, when the symptoms of illness are not present to incentivize adherence. In the current decade, the long-standing barriers against effective tuberculosis diagnosis and treatment are falling. Tools and treatments are increasingly suitable for widespread use in high-incidence settings.

### 1.2. The Contribution of “Subclinical Disease” to Community Transmission

An important element contributing to the existence and magnitude of the tuberculosis pandemic is the rate at which emergent cases are detected and treatment is provided in a community. The WHO estimates that there are 4.1 million people globally with undiagnosed tuberculosis who may be infectious but go unreported to the health system [3]. This estimate is likely to be conservative [37]. There are many reasons why people with tuberculosis do not access care, such as financial and geographical barriers, failure of health services to make a diagnosis, poor diagnostic tools or treatment capacity, and social concerns pertaining to stigma and job loss [38,39,40,41,42]. However, an (until recent times) overlooked factor contributing to under-diagnosis is subclinical tuberculosis, a manifestation of tuberculosis disease that may be detectable through radiology or microbiologic methods (microscope or polymerase chain reaction (PCR)) but does not cause obvious tuberculosis-related symptoms [43]. People with subclinical tuberculosis do not feel ill and thus do not present for care but are still infectious [44,45,46]. Prevalence surveys suggest that subclinical tuberculosis may account for half of all prevalent cases [47,48,49], and modeling estimates indicate that an important majority of global tuberculosis transmission could be from people with subclinical disease [50,51] who may never evince symptoms and continue to mix in communities for long periods of time whilst infectious [52]. Indeed, it is estimated that 80% of transmission occurs before symptoms become apparent (if they ever do), at a time when passive presentation cannot be expected [51]. As most tuberculosis programmes globally rely upon patients to present to health services for care, it is unsurprising that such passive case detection is “missing millions” [51,53,54]. The implications of this are that the timelines for effective intervention before onward transmission are much briefer than previously supposed and will be most important before symptoms appear. ACF strategies where people are screened for tuberculosis using sensitive diagnostic tools, regardless of symptoms, provide an opportunity to detect and treat subclinical and incipient TB, which is otherwise only detected on a small scale in household contact screening [21,55,56,57]. Community-based ACF owes much of its success [23,58] to the way active screening curtails transmission from the dominant 80% fraction of casual community-transmission prior to the symptom-enabled identification of cases. Given this recent understanding of tuberculosis transmission timelines and the prevalence of spread by casual contact, it is challenging to see how other tuberculosis screening strategies (symptom-based, passive, hotspot, close contact) can meaningfully interrupt transmission at a population level and change the epidemiology of the disease.

### 1.3. Persistent Risk Factors for Tuberculosis Infection and Disease

The fact that comparatively few people progress to tuberculosis relative to the vast numbers harboring live *Mtb* bacilli in their lungs demonstrates that non-pathogen factors play an important role in the development of disease and onward transmission [20,59]. Indeed, 95% of all tuberculosis deaths occur in developing countries, and increased risk of disease is associated with crowding and the conditions of poverty that can predominate in such settings [60,61]. Critically, compromised host immune response is also a major risk factor for tuberculosis disease, triggered by smoking, alcohol abuse, very young or very old age [62], air pollution, conflict, refuge-seeking and displacement along with co-morbidities that affect immune fitness such as diabetes and human immunodeficiency virus (HIV) co-infection [8,20,63,64]. Recently, the RATIONS study highlighted the well-known but underestimated consequence of malnutrition on disease risk [65,66]. In this cluster-randomized controlled trial conducted amongst tuberculosis household contacts with prevalent undernutrition, food and macronutrient supplementation was found to be 39% protective against disease progression amongst exposed contacts (incidence rate ratio 0.61 [95% CI 0.43–0.85]) relative to household contacts not receiving food rations [66]. Hailed as “the tuberculosis vaccine we already have” [67], nutritional supplementation for tuberculosis contacts has ethical implications that remain to be fully explored. Selective delivery of food to some but not all households in systemically undernourished communities presents a challenge of justice and community acceptability, which will need to be weighed against the community benefit of reduced tuberculosis disease and transmission. Nevertheless, the RATIONS study challenges a prevailing attitude that tuberculosis interventions targeting social determinants are too distal to measure an effect on disease incidence.

Considering these many factors, settings that combine the opportunity for infection through contact (usually prolonged contact) with a person with infectious tuberculosis [68] with the co-incidence of immunocompromising host and environmental risks are ideal environments for tuberculosis epidemics or endemicity [69,70]. Disease management strategies that are cognizant of these risks and seek to address them, such as screening for diabetes and HIV amongst those with tuberculosis (and vice versa) or the concomitant amelioration of the conditions of poverty, are likely to see better outcomes for patients and communities in the long run [20,71].

### 1.4. The Reservoir of Latent Mycobacterium tuberculosis Infection

One factor contributing to the success of this pathogen is its ability to infect without causing disease. It is estimated that 1.7 billion people—up to one quarter of the global population—is latently infected with *Mtb* [72]. People with latent tuberculosis infection are not usually infectious themselves but represent a large reservoir with heterogenous risk of reactivation from which future cases may develop [73,74]. Current scholarship conceives of latent tuberculosis as a spectrum ranging from stable, long-term quiescent infection with low risk of disease activation to a highly dynamic state with waxing and waning bacilli numbers and active interaction with host immune defence mechanisms in an unstable equilibrium between infection and disease [75,76]. As such, the rigid dichotomy between tuberculosis infection and reactivation is likely to be more fluid than classically conceptualized [77].

In communities with high rates of transmission, it is difficult (and usually irrelevant) to determine whether the disease resulted from the reactivation of an old infection or a recent re-infection event [75,78,79]. However, under an elimination paradigm, the background prevalence of latent infection is highly relevant to ongoing transmission, especially in settings with an ageing demographic [62]. For example, in communities implementing screening programmes for tuberculosis, latent infection may reactivate and transmit disease into screened regions after those with active disease have been detected and treated, undermining the effect and durability of screening upon tuberculosis incidence in the long-term [58]. Similarly, in communities with multi-generational households and ageing populations, the accumulation of risk-factors and immunosenescence in the latently infected elderly may carry an increased risk of reactivation and disease spill-over into younger, more community-mobile household members [62], perpetuating community transmission. This is particularly challenging if risk factors that predisposed communities towards tuberculosis disease progression in the first instance remain in place [20]. Thus, the prevalence of latent infection globally represents a real challenge to tuberculosis elimination and may continue to pose a transmission risk to communities even as disease incidence in younger age groups decline.

Minimizing the impact of latent *Mtb* infection amongst community members through diagnosis and preventive treatment for those exposed also presents challenges. Testing can be time-consuming (tuberculin skin test (TST)) or equipment-intensive (interferon-gamma-release-assay (IGRA)), depending on the setting. The pill burden of treatment (weekly high dose antibiotics for 12 weeks), side-effects, and challenges securing good adherence for people who do not feel ill and are not infectious all contribute to high rates of incomplete treatment [80]. The division between *Mtb* infection and other manifestations of the tuberculosis continuum is also less dichotomous than previously supposed [77]. However, if these challenges can be overcome and combined with community-wide ACF strategies, the simultaneous effect of finding and treating active disease coupled with the elimination of latent *Mtb* reservoirs could have a rapid and durable reduction on community transmission [81]. Further implementation research is required to determine the feasibility of this in each relevant high-incidence setting.

## 2. Community-Wide Active Case Finding for Tuberculosis

*“If we stop testing right now, we’d have very few cases”~Donald Trump*

The value of community-wide ACF has been a topic of discussion and controversy for more than half a century [15,21,55]. As the tuberculosis community approaches the deadlines for ambitious international elimination targets [4,5] with no end to the epidemic in sight, it is clear that current tuberculosis care and prevention strategies have fallen far short of expectations. Even without the devastating impact of coronavirus-19 (COVID-19) upon global tuberculosis management [82,83], the rate of decline in disease incidence has been too slow [84]. Failure to meet targets could be responsible for an avoidable 23.8 million tuberculosis deaths by 2030 and at least USD 11.2 trillion in economic losses globally [85]. Concerningly in some settings, including many climate-threatened island nations of the Asia–Pacific, tuberculosis incidence is on the rise despite accessible passive case detection [86]. In the face of this present and (in some settings) accelerating tuberculosis crisis, a drastic reduction in tuberculosis incidence is urgently needed. The risk of future disease outbreaks and their potential disruptive impact on routine NTP activities, as witnessed during COVID-19 [87], argues for the kind of aggressive intervention that can significantly and rapidly lower tuberculosis incidence—and thus lower the extensive case burden on NTPs—today. Population-wide screening for tuberculosis is considered to be expensive, resource-intensive, and logistically challenging but may be cost-saving over a long time horizon from healthcare and societal perspectives [85,88,89,90]. The impact of these activities in reducing catastrophic costs to people affected by tuberculosis [91,92] is also a powerful incentive to re-evaluate this strategy seriously for use in high-incidence settings in general, and especially in communities where transmission amongst young, economically critical age groups is uncontrolled. Despite the known resource intensity, it is important that funders and national programs do not misconceive community-wide ACF as an unremitting activity that needs to be sustainable for long-term delivery. Through finite, relatively short-term, and aggressive screening, treatment, and prevention, the intent of community ACF is to achieve a step-change in TB control, ultimately lowering the need for ongoing high-levels of screening and investment [93].

Four key considerations for any community-wide active-case-finding strategy are explored below, including the need for symptom-agnostic screening, the value of case detection in advance of timelines for passive presentation, when risk-based screening is appropriate (including who is most vulnerable in high-incidence settings), and what lessons about the effect and limits of community-wide ACF can be garnered from modern randomized controlled trials.

### 2.1. Symptom-Agnostic Detection

In 1974, the WHO recommended that population-wide tuberculosis screening be discontinued as a tuberculosis management strategy [94]. A dominant motivation behind this decision was the observation that most people with *Mtb* bacilli confirmed via smear microscopy have rapid, symptomatic disease and are detected by passive case finding [95,96]. This self-presenting group are likely major contributors to the increased risk of disease amongst household contacts compared to casual contacts, as felt illness forces affected people to cease social mixing and remain within the home [24,97,98]. Nevertheless, in high-incidence settings, ~70% of transmission occurs in the community from casual contact, not from known contacts [99,100,101]. Thus, a major reason for rejecting population-wide ACF was premised upon the incorrect assertion that smear-negative cases are not infectious [102,103] and that most infectious cases rapidly develop symptoms and present for care before extended community mixing. In fact, subclinical disease does have lower smear positivity than symptomatic disease [44,46,49,98,104,105], but the volume of undetected subclinical cases in high-incidence settings and the extended length of time that such cases can be active and infectious for in the community magnifies the population impact of this group on overall transmission [48,50]. Furthermore, a significant proportion of subclinical cases are smear- or Xpert-positive [44,47,49,104], and evidence to suggest that subclinical tuberculosis is uniformly less infectious than symptomatic disease is lacking. Rather, there is some suggestion that the intact lung function of people with subclinical disease could even be more efficient at generating infectious aerosols than those with advanced pathology and impaired lung capacity [106]. Thus, if the detection gap is to be narrowed and the “missing millions” of people with undiagnosed disease are to be found, it is essential to employ strategies that detect all tuberculosis disease, including subclinical cases, through symptom-agnostic ACF methods.

### 2.2. Detection in Advance of Passive Case Finding

An important additional consideration is the fact that many people with symptomatic tuberculosis who do present to the health system are still not diagnosed [39]. In one high-incidence setting, actors posing as patients presenting passively with classic tuberculosis symptoms were correctly managed in only 37% of interactions, even after capacity building in tuberculosis diagnosis had been delivered to health providers in the region [107]. This suggests that even amongst symptomatic tuberculosis cases and in the presence of additional diagnostic training, passive case detection will not reliably diagnose tuberculosis. This is likely due to the fact that tuberculosis symptoms (cough, night sweat, fever, weight loss etc.) are also common in persons without tuberculosis. In this context, ACF has the diagnostic advantage over passive detection, as healthcare providers are searching for tuberculosis cases. Crucially, even if those presenting passively are correctly diagnosed, this is likely too late to have a major impact on community transmission as infection may already have been transmitted to the community members they were likely to infect while feeling well and mixing with the wider population. In contrast to passive detection, the aim of ACF is to reduce the diagnostic delay both at the point of presentation and prior to when this would have occurred during incipience and early disease, thereby increasing the impact of detection on overall transmission.

### 2.3. Targeted Active Case Finding Focused on “Risk Populations”

A common critique of population-wide ACF for tuberculosis is its resource-intensity [56]. Indeed, one motivation behind the 1974 decision by the WHO was that the case yield of population screening had dropped, and such activities were no longer considered to provide value for money [96]. Instead, “hot-spot” or risk-based screening has since predominated [108]. It is certainly the case that population-wide ACF is not readily justifiable in low-burden settings where small and discrete populations with identifiable risk-factors account for most incident tuberculosis [109]. In such contexts, narrow screening amongst communities at risk of disease is likely to be effective, such as amongst migrant populations or people living with HIV [20,59].

From a person-centred care perspective, hot-spot screening amongst vulnerable groups in high-incidence settings will also have immediate positive impacts for affected people and their families, irrespective of its limited impact on overall transmission, e.g., by reducing catastrophic costs, limiting severe adverse effects of disease on the individual, and reducing transmission amongst close contacts. However, from a population perspective, in high-incidence settings, the epidemiology of disease is different, and all people are at risk of disease without belonging to an identifiable risk group [21].

In these epidemiological settings (which are common in the Asia–Pacific), the combination of young populations with ongoing community transmission leads adolescent and young-adult age groups to have some of the highest numbers of incident disease, despite the fact that these groups do not carry the greatest burden of risk-factors. In such settings, risk-based screening within the same communities will fail to make an epidemiological impact on disease incidence because it will tend to exclude the large populations of young people with few risk factors but who bear significant burden of disease and are likely to contribute the most to onward transmission due to enhanced community mobility and social mixing [78,110,111,112].

By consequence, classical directly observed treatment short-course (DOTS) strategies [113] and targeted screening amongst vulnerable populations (especially people living with HIV, diabetes and recently exposed tuberculosis household contacts) will always be a cornerstone of tuberculosis care delivered by national tuberculosis programs (NTPs), but this paradigm of managing tuberculosis is not enough. To make meaningful strides towards eliminating tuberculosis amongst entire populations, NTPs must also be prepared to engage in activities that target population-level vulnerability and should be supported in this (admittedly monolithic) aim by broader country buy-in beyond the health system (considered further below).

### 2.4. Modern Examples of Community-Wide Active Case Finding

Consistent with mid-century findings regarding the effectiveness of population-wide ACF, the ACT3 cluster-randomized controlled trial conducted in a high-incidence setting found that annual door-to-door whole-of-population screening with X-ray and Xpert diagnosis (PCR-based microbiological confirmation) for four years could reduce prevalence of tuberculosis by 44% compared to the non-intervention group (prevalence ratio 0.56, 95% CI 0.40 to 0.78, *p* < 0.001) [23]. This study took place in a rural, low-income, high tuberculosis incidence community in Vietnam and demonstrated the effectiveness of population-wide ACF in reducing tuberculosis transmission in the modern era.

In contrast, an earlier ZAMSTAR enhanced case finding trial that employed community mobile clinics rather than door-to-door care and used smear microscopy diagnosis did not show a difference between intervention and comparison communities [24]. In the latter case, smear negative tuberculosis cases were not included—a significant omission that would have overlooked the majority of subclinical cases [30,102,103]. Furthermore, cases detected by the intervention only accounted for a quarter of all smear-positive cases reported in intervention communities, suggesting that the screening process was not as “aggressive” or community-wide as intended, but was still heavily dependent upon passive case detection [114]. Another important difference between the studies is the variation in sensitivity of diagnostic tools used. Advances in chest X-ray mobility and sputum-based PCR diagnostics employed in ACT3 improved the fitness of diagnostics for use in resource-limited settings. As the availability and affordability of these tools increase, so too do opportunities to implement ACF at scale in settings where less-aggressive elimination strategies have failed.

Nevertheless, lessons learned from the ZAMSTAR study make a critical point: however valuable ACF may be in theory for a high-incidence community, the use of sub-optimal diagnostic tools, poor screening coverage, and/or limited treatment uptake and adherence will undermine the intended effect. Meta-reviews that assess the effect of community-wide ACF interventions on tuberculosis epidemiology tend to include studies that failed to achieve significant population coverage, thereby not meeting pragmatic definitions of “community-wide” ACF. In addition, community-wide screening interventions that fail to adapt to each community in its unique needs, i.e., different levels of crowding, social mobility, use of unventilated public transport, etc., will also limit the effectiveness of “one size fits all” interventions [115]. As a consequence, pooled evidence for the effectiveness of ACF is often inconsistent and of low quality [116]. A nuanced interpretation of the existing evidence might rank studies by their success in truly achieving community-wide case finding, assessing the effect on tuberculosis incidence between studies based upon levels of screening coverage and social mixing. This comparison could also enable the identification of activities that are indispensable in rendering community-wide ACF feasible and implementable, such as structural interventions to introduce ventilation on public transport, as one example [115].

## 3. Implementing What We Know

*“You already have more knowledge than you use.”~François Fénelon*

In response to the above considerations and studies, the WHO revised recommendations for community-wide tuberculosis ACF in 2021, and it currently recommends community-wide ACF in populations with a tuberculosis prevalence of 0.5% or higher and/or in populations with structural risk factors for tuberculosis [108]. Despite this WHO endorsement, uptake of this strategy is still lacking in relevant communities [93]. The wide gulf between what is known (evidence) and what is applied (practice) is mimicked across the medical sciences and has spawned the field of implementation science to develop strategies that close the evidence–practice gap [117]. Implementation science, operational research, and similar evidence-generating activities are siblings to randomized control trials and cohort studies. Where the “high-powered” trial data answer narrow questions with high certainty, e.g., *does community-wide ACF reduce population-level tuberculosis transmission*? Implementation studies are called upon to answer broad questions with low certainty [118], e.g., *how does community-wide ACF work? Why does community-wide ACF work in some communities and not others? What systems need to be in place before community-wide ACF can succeed*?

Without heavy investment in smaller, context-relevant implementation investigations prior to scale-up and launch of community-wide ACF, we risk the delivery of sub-standard ACF that does not achieve community-wide goals and instead entrenches negative views towards tuberculosis screening in the community [119]. The limited roll-out of community-wide ACF beyond the research setting points to NTPs that are either under-resourced and under-supported to implement these WHO guidelines, or are unconvinced that they can be implemented feasibly and effectively within their individual contexts based upon their local evidence and knowledge [120].

Local studies investigating the best methods for implementation and which pave a pathway towards scale-up in each unique setting are needed to attract buy-in from local political actors, NTPs, and communities [120,121,122,123]. Capacity-building endeavors such as the SORT-IT operational research and training have made strides towards bridging this translation-gap [124,125,126], but the critical role of local implementation science in driving forward the tuberculosis elimination agenda requires greater attention from the broader research community and will be critical to maximize the prospects of success in each community [115].

Importantly, this “attention” will likely require a shift amongst funders and researchers to hold local evidence generated in the global South—often published in local journals for consumption by a local audience—of more value than studies available in high-impact journals for consumption by the global North [127]. If NTPs are to be the right hand that moves community-wide ACF forward and reaches towards tuberculosis elimination, then the experiences written by that hand, and the local knowledge existing within such communities, will be of greater significance than WHO policy or international guidelines in the choice of program activities. Funders should be as swayed (if not more so) by the body of local evidence supporting the readiness for community-wide ACF in a community as by the magnitude of international trial evidence, by the credentials of researchers, or by the novelty of the proposed studies.

## 4. Conclusions

In community-wide ACF, the tuberculosis community already has a mechanism with which to interrupt tuberculosis transmission at a population level. The next and unarguably more Herculean challenge is to make what we know works in a trial context work in real life, work better, and work for everyone, irrespective of their cultural, social, geographical, and economic milieu. It is unlikely that grander-scale trials in settings that have already demonstrated the capacity to implement community-wide ACF effectively or more meta-analyses or more directive WHO guidelines will empower local NTPs to any greater degree than the international evidence that already exists. The world can no longer afford to wait (if we ever could) for a more perfect solution to our deadliest pandemic. We must act on what we know. Acquiring local evidence, local knowledge, and funding for small-scale implementation—leading to funding for large-scale implementation—of community-wide ACF is a slow process but represents a meaningful stepping-stone towards using the knowledge we already have to eliminate tuberculosis in high-incidence communities and loose the grip this disease has on our world and its people.

## Data Availability

Not applicable.
